# Dietary Pomegranate By-Product Alleviated the Oxidative Stress Induced by Dexamethasone in Laying Hens in the Pre-Peak Period

**DOI:** 10.3390/ani11041022

**Published:** 2021-04-05

**Authors:** Yahya Eid, Abeer A Kirrella, Ahmed Tolba, Maged El-Deeb, Samy Sayed, Hanan B. El-Sawy, Mustafa Shukry, Mahmoud A. O. Dawood

**Affiliations:** 1 Department of Poultry Production, Faculty of Agriculture, Kafrelsheikh University, Kafrelsheikh 33516, Egypt; yahya.eid@agr.kfs.edu.eg (Y.E.); AKamel@agr.kfs.edu.eg (A.A.K.); 2 Animal Production Research Institute, Agricultural, Research Center, Ministry of Agriculture, Dokki, Giza NC 12618, Egypt; ahmedt@agr.kfs.edu.eg (A.T.); Maged@agr.kfs.edu.eg (M.E.-D.); 3 Department of Science and Technology, University College-Ranyah, Taif University, Ranyah 21975, Saudi Arabia; samy_mahmoud@hotmail.com; 4 Department of Nutrition and Clinical Nutrition, Faculty of Veterinary Medicine, Kafrelsheikh University, Kafr El-Sheikh Governorate, Kafrelsheikh 33516, Egypt; Hanan@agr.kfs.edu.eg; 5 Department of Physiology, Faculty of Veterinary Medicine, Kafrelsheikh University, Kafrelsheikh 33516, Egypt; mostafa.ataa@vet.kfs.edu.eg; 6 Department of Animal Production, Faculty of Agriculture, Kafrelsheikh University, Kafrelsheikh 33516, Egypt

**Keywords:** pre-peak, pomegranate peel, oxidative stress, laying hen production, physiological response

## Abstract

**Simple Summary:**

The present work was designed to maximize an agro-industrial by-products’ benefits in an environmentally safe manner. This study explores the antioxidative effects of pomegranate peels as dietary additives on the performance of laying hens in the pre-peak period under oxidative stress induced by dexamethasone. Pomegranate peel powder was included at 2% and 4% in the diets of laying hens exposed to oxidative stress induced by dexamethasone compared with negative and positive control groups for 12 weeks. Based on the obtained results, the current study recommends the possibility of using dietary pomegranate peels up to 4% not only for alleviating the adverse effects of oxidative stress in the pre-peak laying period but also as a sustainable and economical approach for agricultural development.

**Abstract:**

This experiment was conducted to assess the inclusion of the by-products of pomegranate peels to ameliorate the harmful impacts of oxidative stress in the pre-peak period of laying hens. For this, 120 local Egyptian strain hens (Inshas hens) aged 24 weeks old were used in four treatments. Pomegranate peel powder was included at 2% and 4% in the diets of laying hens subjected to oxidative injuries induced by dexamethasone compared with negative and positive control groups for 12 weeks. The addition of pomegranate peel powder (PPP) reduced the adverse effects of oxidative stress induced by dexamethasone on body weight (*p* = 0.006) and egg production (*p* = 0.010) comparing to the positive control. Additionally, pomegranate peel powder had a significant positive lowering effect on plasma cholesterol (*p* < 0.001) and triglyceride contents (*p* = 0.005) compared to control groups. The lipid peroxidation indicators (MDA) were reduced, but the antioxidative enzymes (SOD, CAT, and GPx) and total antioxidant blood capacity were improved with PPP. Based on the obtained data, the present research recommends using dietary PPP up to 4% to mitigate adverse oxidative stress effects in the pre-peak laying period and as a sustainable and economical approach for agricultural development.

## 1. Introduction

Sexual maturity and the beginning of the laying period are critical for laying hens, and may negatively impact hens’ performance [1]. The pre-peak period is a physically challenging period for young chickens as nutrient deficiencies can affect liver and bone metabolism [2].

The twelve ecological development goals of the United Nations (https://www.un.org/sustainabledevelopment/ accessed on March 2021) encouraged several countries, including Egypt, which has many local inferred strains preserved for the use of local genetic resources. These strains require continuous improvement and development of care methods, which can be deployed in small to medium poultry projects. However, these lineages may be less productive than commercial breeds even though strains are more adapted to the environmental conditions than commercial ones. The pre-peak period may negatively affect the whole production period due to oxidative stress.

Animal stress occurs when changes in the environment happen and are associated with body responses to reestablish homeostatic conditions [3]. Recently, multiple climatic changes and increased populations induced different stress problems (technological, environmental, nutritional, etc.) in birds. Eventually, stress conditions can lead to oxidative stress (OS) [4]. These biotic or abiotic stressors are responsible for adverse effects, including the lack of productive and reproductive performance, and may end with birds’ deaths. Oxidative stress-induced apoptosis in follicular cells leads to reduced follicle numbers and then egg production [5]. On the other hand, stress adversely affects egg quality traits, yolk lipids, and cholesterol contents [3], while many studies reported the beneficial effects of antioxidants on egg quality [3,6,7]. The adverse effects of these stressors cause corticosterone release from the adrenal gland to the plasma in birds. Among glucocorticoids, dexamethasone (DEX) can alleviate corticosterone impacts and cause oxidative stress and immunosuppression if administered at 0·2 to 4·0 mg/kg [8].

Using agro-industrial by-products in poultry feed has received special attention due to the intensification of food production, leading to the generation of large quantities of by-products [7]. Some of these materials are used as feed additives that may have a good effect on the final product in terms of shelf life [6], sensory characteristics (appearance and quality), and nutritional value, and even reduce the cost of nutrition [9]. Pomegranate (*Punica granatum*) is one of the oldest edible fruit crops cultivated widely in many countries, including Egypt [6]. Global production and pomegranate consumption have increased dramatically in recent years regarding the effects of its various components on human health [10]. Pomegranate fruits consist of seeds (about 30% of the weight of the fruit) and peels (up to 60%) [11]. Pomegranate peel powder (PPP) is a rich source of tannins, flavonoids, and phenolic compounds with antioxidant properties [12]. Dietary PPP can improve the digestion and metabolism of fatty acids (oleic, palmitic, stearic, palmitoleic, arachidonic, lauric, and caprylic) [13]. Additionally, PPP has antioxidant [14], immunomodulatory [15], antibacterial [16], and anti-atherosclerotic activity [17].

The present experimental work was designed to serve the sustainable development UN goals by maximizing the use of genetic resources and re-use of agro-industrial by-products in an environmentally safe manner. This study explores the antioxidative effects of pomegranate peels as a dietary component and antioxidant source on the performance of laying hens in the pre-peak period under oxidative stress conditions.

## 2. Materials and Methods

### 2.1. Birds, Diets, and Experimental Design

The experiments were performed according to the guidelines of a local ethics committee (Number 4/2016 EC) at the faculty of Agriculture, Kafrelsheikh University, Kafrelsheikh, Egypt. The authors specifically state that the procedures were carried out in compliance with Directive 2010/63/EU of the European Parliament and the Council of 22 September 2010.

One hundred and twenty birds of the local Egyptian strain Inshas, aged 24 weeks old, were used in this study. The average age of sexual maturity for Inshas hens is 22–25 weeks of age [18]. At the start of the experiment, all birds were weighed individually to the nearest gram and then distributed into four groups and randomly replicated. Each group (30 birds) was randomly split into three replicates; each replicate had ten birds housed in individual battery cages under the same management and environmental conditions (280 cm long × 220 cm wide × 40 cm tall) fixed in an open-sided laying house. The birds were exposed to continuous daily lighting for 17 h. Tap water and experimental mash diets were supplied ad libitum. The experimental period lasted 12 weeks (September to November), and the temperature and relative humidity were 29 °C and 63.3%, respectively.

### 2.2. Materials

Pomegranate peels were obtained from the local juice production factory. For PPP preparation, the peels were washed, air-dried until a constant weight, and powdered to pass through a 40-mesh sieve. After that, the PPP was packed and stored at 8 ± 1 °C. The chemical composition of the pomegranate peel powder (% of dry weight) was evaluated (Table 1) according to Association of Official Analytical Chemists (AOAC) [19].

The phytochemical constituents of pomegranate peel were assayed. Total phenols, flavonoids, and antioxidant activity were estimated according to El-Hadary and Taha [6] (Table 2).

The oxidative stress was caused by the synthetic steroid dexamethasone (DEX) (36.4 mg/kg diet) for T2, T3, and T4 groups throughout the experimental period, where each hen received 4 mg/kg body weight with the average daily feed consumption according to Eid et al. [20]. DEX was purchased from the El-Kahira pharmaceuticals and chemical industry company (Shoubra, Elsahel, Cairo Governorate, Egypt).

### 2.3. Experimental Design

Birds were randomly divided into four treatments (*n* = 30 birds for each treatment). T1 (negative control) was fed a basal diet without oxidative stress. All remaining groups were exposed to oxidative stress without pomegranate peel powder (PPP) feeding (T2, positive control) or with feeding of PPP at 2% (T3) and 4% (T4), as presented in Table 1. Experimental diets were formulated to cover the hens’ nutritional needs, as described by National Research Council (NRC) [21].

### 2.4. Performance Indicators

Body weight gain (BWG), average daily feed intake (ADFI), and egg production (%, EP) were recorded individually for 12 weeks. In addition, egg mass (number of eggs × average weight of eggs) and feed conversion ratio (g feed consumed/g egg mass) were also calculated. All traits were measured weekly and are presented as an average of the experimental period.

### 2.5. Egg Quality

At the end of the experiment, a total of 20 fresh laid eggs/treatments were randomly taken from each treatment on the same day. Egg quality measurement (i.e., egg weight, width, length, yolk weight, albumen weight, shell weight, albumen, yolk height, eggshell thickness, yolk percent, yolk index, egg shape index, and Haugh units (HUs)) values were determined according to Keshavarz and Nakajima [22]. At first, eggs were weighed individually, then widths and lengths were measured using the same Vernier caliper. After that, eggs were broken on a smooth level surface to determine egg quality traits using the following tools: digital sensitive scales (egg weight, yolk weight, albumen weight, and shell weight), steel Vernier caliper (egg length and width and yolk diameter), tripod micrometer (albumen and yolk height), and an “Ames” micrometer (eggshell thickness). From the previous measurements, the following quality measures were calculated: yolk percent, yolk index, egg shape index, and Haugh unit (HU) values, which were calculated as follows:

HU = 100 log (h + 7.57 − 1.7 W0.37)

where H is the albumen height (mm) and W is the egg weight (g).

Yolk total lipids and cholesterol were measured according to Folch et al. [23] and Rudel and Morris [24], respectively. In this process, 15 mL of 2:1 chloroform–methanol solution were added to 1 gm yolk; after that, the sample was shaken by hand, 5 mL of distilled water were added, hand shaken again and then centrifuged. After that, the aqueous methanol layer was eliminated, and the chloroform layer was filtered to assess total lipids and cholesterol content by chromatographic assay.

### 2.6. Blood Sampling

At 36 weeks of age, ten hens per treatment were randomly chosen, then slaughtered within 1–2 h post-oviposition (at 2 pm), and blood samples for individuals were collected into heparinized tubes. The blood sample was centrifuged at 3000× *g* for 15 min to separate blood plasma (*n* = 10/treatment), then kept at –20 °C for the evaluation of plasma total cholesterol [25], high-density lipoprotein (HDL) cholesterol [26], low-density lipoprotein (LDL) cholesterol [27], triglycerides [28], malondialdehyde (MAD) [29], antioxidant enzymes (superoxide dismutase (SOD), catalase (CAT), and glutathione peroxidase (GPx)) [30,31] and total antioxidant capacity [32] by using Biomerieux Kits (Biomerieux, Marcy l’Etoile, France) following the manufacturer’s guidelines. The samples were analyzed separately for the indices mentioned above.

### 2.7. Data Analysis

The analysis was conducted using a one-way ANOVA by SPSS^®^ statistical software version 15. A significant difference among treatment means was detected by Duncan’s multiple range test at a 5% level of significance (*p* ≤ 0.05). The impact of pomegranate peels under oxidative injury on the production and physiological parameters of laying hens was studied by applying the following model:

Yij = M + Ti + eij

where:

Yij = observation for each dependent variable.

M = overall mean.

Ti = treatment effects (I = 1, 2, 3, 4).

eij = random error.

## 3. Results

The productive characteristics of laying hens are presented in Table 3. There were no significant differences in the initial body weight at the beginning of the experiment. The inclusion of dietary PPP at 4% with DEX resulted in a higher final body weight (*p* = 0.007), body weight gain (*p* = 0.006), average daily feed intake (*p* = 0.007), egg mass (*p* = 0.001), egg production (*p* = 0.01), and weight (*p* = 0.001), in addition to lower feed conversion ratio (*p* = 0.019), than the positive control.

In terms of egg quality traits (Table 4), the yolk weight (YW) (*p* = 0.006) and shell weight (SW) (*p* = 0.001) showed a reduced value in the group of birds treated with DEX when compared to the other groups. Additionally, the albumen weight (AW) had lower values in the groups treated with DEX without PPP or 2% PPP (*p* = 0.044). The shell thickness (ST) had a higher value in the group of birds fed dietary PPP at 4% than those fed a PPP-free diet with or without DEX, while the group treated with 2% PPP showed no substantial changes compared to the negative control group (*p* = 0.006).

**Figure 1:**
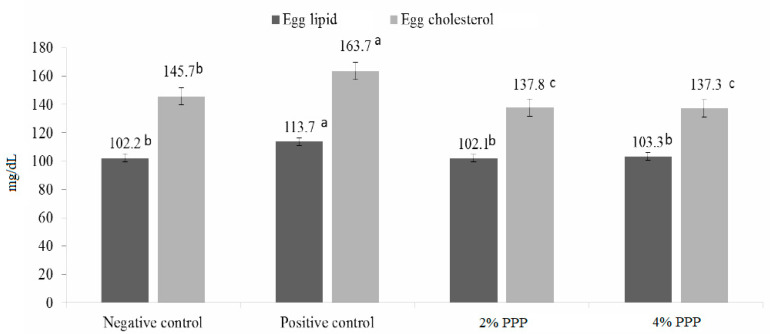
Effect of different levels of dietary pomegranate peel on egg yolk lipid content (*p* ˂ 0.001) and cholesterols (*p* ˂ 0.001) of laying hens subjected to oxidative stress. Bars sharing a common superscript are not statistically different. DEX, dexamethasone; PPP, pomegranate peel powder (*n* = 10 birds for each treatment). ^a–c^ Bars with different superscript are significantly different (*p* ≤ 0.05).

As for the chemical content of eggs (Figure 1), the use of PPP significantly reduced the egg content of cholesterol (*p* = 0.001) and lipids (*p* = 0.001) compared to laying hens exposed to oxidative stress and was superior to that in non-stressed chickens, where the treatment supplemented with 4% PPP achieved the best values of 103.3 g and 137.3 mg/dL, respectively.

According to Table 5, dietary PPP led to a noteworthy reduction in the plasma content of total cholesterol (*p* < 0.001), LDL cholesterol (*p* < 0.001), and triglycerides (*p* = 0.005), while causing a significant increase in HDL cholesterol (*p* < 0.001) in the treatment that was subjected to oxidative stress.

The data presented in Table 6 show a significant reduction in the concentration of antioxidant enzymes (SOD, CAT, and GPx) and total antioxidant capacity (*p* < 0.001). However, there was an upsurge in the blood plasma content of MDA (*p* < 0.001) in the positive control group that was subjected to oxidative stress. All of these adverse effects were significantly improved by using pomegranate peels in the laying hens’ diets.

## 4. Discussion

It is known that stress is the body’s unspecified biological response as a reaction to harmful stimuli, which negatively affects normal physiological balance [3]. There are many stressful factors, including thermal radiation, air temperature, sunlight, humidity, movement, etc. [33]. Oxidative stress is the result of all stressors, and it is also a significant adverse consequence for the most common commercial agents in poultry production [4]. Such negative impacts of oxidative stress could lead to significant economic losses in poultry laying farms, especially in the pre-peak period of laying, which may have long-lasting adverse effects across the production period. In the current study, birds exposed to oxidative stress using dexamethasone had lower pre-peak productive performance than the control. The generated free radicals resulting from oxidative stress cause damage to many biocompounds in the birds, such as fats, carbohydrates, nucleic acids, and proteins, thus changing their biological function [34]. These free radicals negatively affect feed intake, digestion, and nutrient absorption by damaging the intestinal mucosa because of oxidation impeding the digestion and absorption of useful nutrients and negatively affect natural growth [35]. Including PPP in the hens’ diet successfully ameliorates these adverse effects. This may refer to its contents of antioxidants, substances, and fiber, which reduce the attacks of free radicals in the digestive tract and all body organs and may enhance the palatability of diets, which had better FI, FCR, and performance.

The decline in the production of eggs in our research may be attributed to a reduction in feed consumption., which reduces the availability of the materials needed to produce eggs. An increase in glucocorticoid levels in the blood leads to blocked ovulation, which leads to suppressed luteinizing hormone (LH) and progesterone production, as well as prostaglandin, and also reduces the inhibin production of granulosa cells, which in turn affects the growth and development of egg sacs by affecting pituitary follicle-stimulating hormone (FSH) [36]. Recently, the poultry industry has turned to phytochemicals and herbaceous plants to minimize adverse stress and increase poultry performance [37]. The use of PPP in poultry feeding has been studied in several studies that were compatible with the current study, all of which emphasized the good effects of pomegranate by-products on production rates, either under normal or stress conditions [38]. The main compounds in pomegranate peels are phenolic compounds, such as punicalagin, gallic acid, fatty acids, catechin, quercetin, rutin, flavonols, flavones, flavanones, and anthocyanidins [39]. Additionally, PPP comprises 67% of the total phenolic content in whole fruit [40]; and the large amount of phenolics may cause its strong antioxidant ability [41]. Among the most critical factors that may explain pomegranate peel’s positive effects are palatability and digestion, its effect on intestinal enzymatic activity, and increased digestion and absorption [42]. Further, it can improve immune properties, activate enzymatic antioxidants, and control free radicals during stress conditions due to antioxidant, antimicrobial, anti-inflammatory, and anticancer properties [43]. This helps to reduce pathogens in the intestine; thus, additional nutrients are accessible in the intestinal cavity to be absorbed to convert into body mass [44].

It is stated that high levels of circulating glucocorticoids resulted in a decrease in egg production [45], which was confirmed previously by Eid et al. [20]. DEX administration stopped ovulation and suppressed LH and progesterone production [46]. Additionally, DEX induced a significant decrease in the production of prostaglandin E2 and lowered inhibin generation by granulosa cells in vitro, which lowered the concentration of inhibin in plasma [46]. Inhibin, a dimeric glycoprotein, is essential for controlling follicular development and negatively impacts the secretion of pituitary FSH [47]. DEX can accumulate in the yolk-filled preovulatory follicles and indirectly inhibit the production of inhibin by its surrounding granulosa layer [48]. These mechanisms can account for the negative impact on the production of eggs caused by glucocorticoids (DEX).

Eggs are known to consist of water (74%), proteins (12%), lipids (12%), carbohydrates (<1%), vitamins, and minerals [49]. In agreement with our study, oxidative stress has been reported to cause significant adverse effects on egg quality traits. It negatively affects metabolic levels, which reduces the opportunity to provide these substances, which may reduce the internal and external egg quality features, such as the yolk properties, albumin, and Haugh units [13]. Along the same line, this may be due to the effect of DEX on digesting and absorbing the nutrients necessary for egg production. Moreover, the reduction in cholesterol and triglycerides by adding pomegranate peels most likely owed to its high content of flavonoids and tannins, which have high antioxidant activity [13].

Results related to levels of cholesterol and triglycerides in the blood, obtained in the current study, were consistent with other studies [13], which found a decrease in the level of fats and glucose as a result of feeding birds pomegranate peels, which may be due to the high percentage of fiber [50], which is associated with bile salts in the intestine, reduces intestinal transit time, and increases bile secretion. Hence, it increases sterol metabolism, leading to low cholesterol in the blood [13,51]. Lower cholesterol in the blood and triglycerides by PPP could possibly be due to phenol compounds (pontiacagen, pontiacin, gallic acid, and allicin acid in particular) [6]. Moreover, it may stimulate pomegranate polyphenols and promote cholesterol metabolism by modifying HDL transport [52].

The results of the current study agreed with many studies on pomegranate peels’ ability to improve rates of the production of antioxidant enzymes and the total antioxidant capacity in birds under stress conditions in addition to reducing MDA [43]. The release of free radicals in the blood of birds in large quantities under stress conditions reduces anti-oxidation enzymes, such as GPx, SOD, and CAT, which are considered the main antioxidant enzymes in the cell and are associated with free radical detoxification. The observed effectiveness of pomegranate powder and extracts in plasma-measured oxidation indices may be due to phenolic compounds’ role in the inhibition of the oxidation process in plants [4].

Several investigations have demonstrated that oxidative stress caused damage in a variety of tissues in poultry. These pathological changes included fatty degeneration, with the enlargement of sinusoid and necrosis, and with heterophils and lymphocytes in cells [53] and hyperemic blood vessels [54]. Antioxidants have been shown to activate ROS and thus protect cells from damage [55]; this may explain the positive effects of PPP in reducing liver and spleen cell damage as it contains a large number of antioxidant compounds [39,40].

## 5. Conclusions

Based on the results mentioned above, the addition of pomegranate peel powder to laying hens’ diets, especially in the pre-peak period, alleviated the adverse effect of oxidative stress on body weight, egg production, and egg quality characterictics, as well as had a significant positive effect on biochemical blood variables and the plasma content of cholesterol and triglycerides. The same trend was observed for antioxidative enzymes and the total antioxidant capacity of the blood. On the other hand, physiological damage decreased in the spleen and liver tissues. Such a sustainable approach of using agro-industry by-products may positively impact performance indicators and enhance the physiological systems of the laying hens under oxidative stress, especially during the pre-peak period.

## Figures and Tables

**Table 1:** Composition and calculated chemical analysis of the basal experimental diet.

Item	Control	Pomegranate Peel Powder	
		2%	4%
Yellow corn	66	63.6	61.2
Soybean meal (44%)	24	24.3	24.6
Wheat bran	0	0.1	0.2
PPP	0	2	4
Limestone	7.59	7.59	7.59
Di-calcium phosphate	1.71	1.71	1.71
Sodium chloride	0.3	0.3	0.3
Vit. and min. mixture *	0.3	0.3	0.3
DL. Methionine	0.1	0.1	0.1
Total	100	100	100
Calculated analysis			
Metabolizable energy (MJ/kg)	2750	2751	2751
Crude protein, %	16.43	16.43	16.43
Crude fiber, %	3.20	3.43	3.66
Ether extract, %	2.70	2.65	2.61
Calcium, %	3.33	3.40	3.47
Available phosphate, %	0.45	0.47	0.50
Lysine, %	0.39	0.39	0.38
Methionine, %	16.43	16.43	16.43

* Supplied per kg of diet: vit. A, 10,000 IU; D_3_, 2000 IU; vit. E, 10 mg; vit. K_3_,1 mg; vit. B_1_, 1 mg; vit. B_2_, 5 mg; vit. B_6_, 1.5 mg; vit. B_12_, 10 mcg; niacin, 30 mg; pantothenic acid, 10 mg; folic acid, 1 mg; biotin, 50 µg; choline, 260 mg; copper, 4 mg; iron; 30 mg; manganese, 60 mg; zinc, 50 mg; iodine, 1.3 mg; selenium, 0.1 mg; cobalt, 0.1 mg.

**Table 2:** Chemical composition of pomegranate peel powder (% on dry weight basis).

Items	Components %
DM	94.79
CP	3.29
EE	1.93
CF	12.48
Ash	3.84
TP	30.98
TF	25.59
AA	11.54

The chemical composition of pomegranate peel powder (% of dry weight) and phytochemical constituents represent the mean of triplicate determination results (*n* = 3). DM, dry matter; CP, crude protein; EE, ether extract; CF, crude fiber; TP, total phenols; TF, total flavonoids; AA, antioxidant activity; Ash, minerals.

**Table 3:** Effect of different levels of dietary pomegranate peel on the performance of laying hens subjected to oxidative stress.

Production Traits	Negative Control (−DEX)	Positive Control (+DEX)	2% (PPP + DEX)	4% (PPP + DEX)	*p*-Value
IBW (g)	1333 ± 0.520	1333 ± 1.322	1331 ± 0.784	1333 ± 1.622	0.548
FBW (g)	1554 ± 21.16 ^ab^	1468 ± 13.19 ^c^	1536 ± 17.67 ^b^	1599 ± 20.82 ^a^	0.007
BWG (g)	221 ± 21.09 ^a^	135 ± 11.92 ^b^	205 ± 18.44 ^a^	266 ± 20.04 ^a^	0.006
ADFI (g/d)	92.5 ± 0.407 ^a^	83.2 ± 0.758 ^b^	88.6 ± 1.035 ^a^	90.1 ± 2.349 ^a^	0.007
EP (%)	52.79 ± 1.834 ^a^	47.49 ± 1.442 ^b^	54.14 ± 1.218 ^a^	56.43 ± 0.687 ^a^	0.010
EW (g)	47.14 ± 0.077 ^a^	43.32 ± 0.90 ^c^	45.26 ± 0.126 ^b^	46.82 ± 0.783 ^a^	0.001
FCR (g fed/g egg)	3.72 ± 0.113 ^ab^	4.05 ± 0.155 ^a^	3.61 ± 0.039 ^b^	3.41 ± 0.095 ^b^	0.019
EM (g/hen/day)	24.88 ± 0.825 ^a^	20.62 ± 0.658 ^b^	24.50 ± 0.546 ^a^	26.41 ± 0.182 ^a^	0.001

DEX, dexamethasone; PPP, pomegranate peel powder; IBW, initial body weight; FBW, final body weight, BWG, body weight gain; ADFI, average daily feed intake; EP, egg production %; EW, egg weight; FCR, feed conversion ratio; EM, egg mass. ^a–c^ Means within a column sharing a common superscript are not different (*p* ≤ 0.05) (*n* = 30 birds for each treatment).

**Table 4:** Effect of different levels of dietary pomegranate peel on egg quality of laying hens subjected to oxidative stress.

Parameter	Negative Control (−DEX)	Positive Control (+DEX)	2% (PPP + DEX)	4% (PPP + DEX)	*p*-Value
EL (mm)	55.4 ± 1.17 ^a^	53.0 ± 1.44 ^a^	55.1 ± 0.44 ^a^	55.3 ± 0.35 ^a^	0.315
ED (mm)	44.7 ± 0.40 ^a^	41.1 ± 2.23 ^a^	43.9 ± 0.97 ^a^	43.6 ± 0.68 ^a^	0.291
ESI	80.8 ± 1.23 ^a^	77.6 ± 4.20 ^a^	79.7 ± 2.29 ^a^	78.9 ± 1.68 ^a^	0.850
YW (g)	15.8 ± 0.29 ^a^	14.2 ± 0.31 ^b^	15.6 ± 0.40 ^a^	16.7 ± 0.37 ^a^	0.006
AW (g)	25.9 ± 0.24 ^a^	24.1 ± 0.43 ^b^	24.4 ± 0.4 ^b^	24.8 ± 0.47 ^ab^	0.044
YH (mm)	17.7 ± 0.28 ^a^	17.2 ± 0.11 ^a^	17.9 ± 0.43 ^a^	17.6 ± 0.34 ^a^	0.541
YD (mm)	41.5 ± 0.83 ^a^	40.1 ± 0.30 ^a^	41.5 ± 0.66 ^a^	40.7 ± 0.57 ^a^	0.373
YI	42.6 ± 1.53^a^	42.9 ± 0.52 ^a^	43.0 ± 0.71 ^a^	43.2 ± 1.11 ^a^	0.955
AH (mm)	5.83 ± 0.06 ^a^	5.65 ± 0.27 ^a^	6.39 ± 0.30 ^a^	5.81 ± 0.30 ^a^	0.254
HU (%)	80.1 ± 0.42 ^a^	80.3 ± 1.82 ^a^	84.4 ± 1.92 ^a^	80.0 ± 1.83 ^a^	0.240
SW (g)	5.35 ± 0.05 ^a^	5.04 ± 0.04 ^b^	5.30 ± 0.04 ^a^	5.35 ± 0.01 ^a^	0.001
ST (mm)	0.35 ± 0.003 ^b^	0.34 ± 0.007 ^c^	0.37 ± 0.013 ^ab^	0.38 ± 0.009 ^a^	0.006

DEX, dexamethasone; PPP, pomegranate peel powder; EL, egg length; ED, egg diameter; ESI, egg shape index; YW, yolk weight; SW, shell weight; AW, albumen weight; YH, yolk height; YD, yolk diameter; YI, yolk index; SW, shell weight; ST, shell thickness; AH, albumen height; and HU, Haugh units. ^a–c^ Means within a column sharing a common superscript are not different (*p* ≤ 0.05) (*n* = 20 birds for each treatment).

**Table 5:** Effect of different levels of dietary pomegranate peel on some biochemical parameters of laying hens subjected to oxidative stress.

Blood Biochemical Traits	Negative Control (−DEX)	Positive Control (+ DEX)	2% (PPP + DEX)	4% (PPP + DEX)	*p*-Value
CH (mg/dL)	158.80 ± 0.352 ^b^	189.20 ± 1.189 ^a^	146.77 ± 0.890 ^c^	145.42 ± 0.763 ^c^	<0.001
HDL (mg/dL)	98.18 ± 0.46 ^a^	79.94 ± 0.50 ^c^	92.83 ± 0.57 ^b^	99.25 ± 0.52 ^a^	<0.001
LDL (mg/dL)	33.00 ± 0.80 ^b^	41.70 ± 0.26 ^a^	32.35 ± 0.20 ^b^	32.05 ± 0.17 ^b^	<0.001
TG (mg/dL)	293.15 ± 19.14 ^b^	337.86 ± 8.88 ^a^	270.16 ± 5.78 ^b^	259.49 ± 6.36 ^b^	0.005

DEX, dexamethasone; PPP, pomegranate peel powder; CH, cholesterol; HDL, high-density lipoprotein; LDL, low-density lipoprotein; TG, triglycerides. ^a–c^ Means within a column sharing a common superscript are not different (*p* ≤ 0.05) (*n* = 10 birds for each treatment).

**Table 6:** Effect of different levels of dietary pomegranate peel on antioxidative indicators of laying hens subjected to oxidative stress.

Antioxidative traits	Negative Control (−DEX)	Positive Control (+ DEX)	2% (PPP + DEX)	4% (PPP + DEX)	*p*-Value
MDA (nmole/L)	9.21 ± 0.28 ^b^	14.17 ± 0.43 ^a^	9.40 ± 0.30 ^b^	9.50 ± 0.43 ^b^	<0.001
SOD (U/mL)	121.12 ± 1.00 ^a^	94.52 ± 0.72 ^c^	110.88 ± 0.97 ^b^	120.89 ± 1.08 ^a^	<0.001
GPx (U/L)	1.58 ± 0.04 ^b^	1.29 ± 0.42 ^c^	1.70 ± 0.62 ^ab^	1.83 ± 0.59 ^a^	<0.001
CAT (U/mL)	7.45 ± 0.16 ^b^	6.00 ± 0.24 ^c^	7.19 ± 0.07 ^ab^	7.41 ± 0.27 ^a^	<0.001
TAC (mmole/L)	0.44 ± 0.01 ^b^	0.29 ± 0.03 ^c^	0.50 ± 0.01 ^ab^	0.53 ± 0.02 ^a^	<0.001

DEX, dexamethasone; PPP, pomegranate peel powder; MDA, malondialdehyde; GPx, glutathione peroxidase; SOD, superoxide dismutase; CAT, catalase; TAC, total antioxidant capacity. ^a–c^ Means within a column sharing a common superscript are not different (*p* ≤ 0.05) (*n* = 10 birds for each treatment).

## References

[1] Sujatha T., Rajini R.A., Prabakaran R. (2014). Efficacy of pre-lay diet. J. Appl. Anim. Res..

[2] Woelders H., Zuidberg C.A., Hiemstra S.J. (2006). Animal genetic resources conservation in the Netherlands and Europe: Poultry perspective1. Poultr. Sci..

[3] Scanes C.G. (2016). Biology of stress in poultry with emphasis on glucocorticoids and the heterophil to lymphocyte ratio. Poultr. Sci..

[4] Surai P.F., Kochish I.I., Fisinin V.I., Kidd M.T. (2019). Antioxidant defence systems and oxidative stress in poultry biology: An update. Antioxidants.

[5] Li G.-M., Liu L.-P., Yin B., Liu Y.-Y., Dong W.-W., Gong S., Zhang J., Tan J.-H. (2020). Heat stress decreases egg production of laying hens by inducing apoptosis of follicular cells via activating the FasL/Fas and TNF-α systems. Poultr. Sci..

[6] El-Hadary A.E., Taha M. (2020). Pomegranate peel methanolic-extract improves the shelf-life of edible-oils under accelerated oxidation conditions. Food Sci. Nutr..

[7] Sugiharto S., Yudiarti T., Isroli I., Widiastuti E. (2018). The potential of tropical agro-industrial by-products as a functional feed for poultry. Iran. J. Appl. Anim. Sci..

[8] Eid Y., Ebeid T., Younis H. (2006). Vitamin E supplementation reduces dexamethasone-induced oxidative stress in chicken semen. Br. Poult. Sci..

[9] Kasapidou E., Sossidou E., Mitlianga P. (2015). Fruit and vegetable co-products as functional feed ingredients in farm animal nutrition for improved product quality. Agriculture.

[10] Avazeh A., Adel M., Shekarabi S.P.H., Emamadi H., Dawood M.A., Omidi A.H., Bavarsad M. (2020). Effects of dietary pomegranate peel meal on the growth performance, blood indices, and innate immune response of rainbow trout (*Oncorhynchus mykiss*). Ann. Anim. Sci..

[11] Ismail T., Sestili P., Akhtar S. (2012). Pomegranate peel and fruit extracts: A review of potential anti-inflammatory and anti-infective effects. J. Ethnopharmacol..

[12] Ibrahim M., Arafa S.A., Hassan F.A., Zaki E.E. (2017). Utilization of pomegranate (*Punica granatum* L.) by-product powder as a natural growth promoter in growing rabbit diets. Egypt. J. Rabbit. Sci..

[13] Abbas R.J., Al-Salhie K.C.K., Al-Hummod S. (2017). The effect of using different levels of pomegranate (*Punica granatum*) peel powder on productive and physiological performance of Japanese quail (*Coturnix coturnix japonica*). Livest. Res. Rural Dev..

[14] Al-Zoreky N.S. (2009). Antimicrobial activity of pomegranate (*Punica granatum* L.) fruit peels. Int. J. Food Microbiol..

[15] Gracious Ross R., Selvasubramanian S., Jayasundar S. (2001). Immunomodulatory activity of *Punica granatum* in rabbits—a preliminary study. J. Ethnopharmacol..

[16] Navarro V., Villarreal M.L., Rojas G., Lozoya X. (1996). Antimicrobial evaluation of some plants used in Mexican traditional medicine for the treatment of infectious diseases. J. Ethnopharmacol..

[17] Tzulker R., Glazer I., Bar-Ilan I., Holland D., Aviram M., Amir R. (2007). Antioxidant activity, polyphenol content, and related compounds in different fruit juices and homogenates prepared from 29 different pomegranate accessions. J. Agric. Food Chem..

[18] El-Ghar A., Sh R., El-Karim A. (2016). Effect of early selection for body weight, keel length and breast circumference on egg production traits in inshas strain of chickens. Egypt. Poult. Sci. J..

[19] AOAC (1999). Association of Official Analytical Chemists, Official Methods of Analysis of AOAC International.

[20] Eid Y., Ebeid T., Moawad M., El-Habbak M. (2008). Reduction of dexamethasone-induced oxidative stress and lipid peroxidation in laying hens by dietary vitamin E supplementation. Emir. J. Food Agric..

[21] NRC (1994). National Research Council. Nutrient Requirements of Poultry.

[22] Keshavarz K., Nakajima S. (1995). The effect of dietary manipulations of energy, protein, and fat during the growing and laying periods on early egg weight and egg components1. Poultr. Sci..

[23] Folch J., Lees M., Stanley G.S. (1957). A simple method for the isolation and purification of total lipides from animal tissues. J. Biol. Chem..

[24] Rudel L.L., Morris M. (1973). Determination of cholesterol using o-phthalaldehyde. J. Lipid Res..

[25] Richmond W. (1973). Preparation and properties of a cholesterol oxidase from nocardia sp. and its application to the enzymatic assay of total cholesterol in serum. Clin. Chem..

[26] Finley P.R., Schifman R.B., Williams R.J., Lichti D.A. (1978). Cholesterol in high-density lipoprotein: Use of Mg2+/dextran sulfate in its enzymic measurement. Clin. Chem..

[27] Friedewald W.T., Levy R.I., Fredrickson D.S. (1972). Estimation of the concentration of low-density lipoprotein cholesterol in plasma, without use of the preparative ultracentrifuge. Clin. Chem..

[28] Fossati P., Prencipe L. (1982). The determination of triglyceride using enzymatic methods. Clin. Chem..

[29] Nair V., Turner G.A., Offerman R.J. (1984). Novel adducts from the modification of nucleic acid bases by malondialdehyde. J. Am. Chem. Soc..

[30] Misra H.P., Fridovich I. (1972). The role of superoxide anion in the autoxidation of epinephrine and a simple assay for superoxide dismutase. J. Biol. Chem..

[31] Aebi H. (1984). [13] Catalase in vitro. Methods Enzymol.

[32] Miller N.J., Rice-Evans C., Davies M.J., Gopinathan V., Milner A. (1993). A novel method for measuring antioxidant capacity and its application to monitoring the antioxidant status in premature neonates. Clin. Sci..

[33] Lara L.J., Rostagno M.H. (2013). Impact of heat stress on poultry production. Animals.

[34] Lv Z.P., Peng Y.Z., Zhang B.B., Fan H., Liu D., Guo Y.M. (2018). Glucose and lipid metabolism disorders in the chickens with dexamethasone-induced oxidative stress. J. Anim. Physiol. Anim. Nutr..

[35] Yara S., Lavoie J.-C., Beaulieu J.-F., Delvin E., Amre D., Marcil V., Seidman E., Levy E. (2013). Iron-ascorbate-mediated lipid peroxidation causes epigenetic changes in the antioxidant defense in intestinal epithelial cells: Impact on inflammation. PLoS ONE.

[36] Tetsuka M. (2007). Actions of glucocorticoid and their regulatory mechanisms in the ovary. Anim. Sci. J..

[37] Saeed M., Babazadeh D., Arif M., Arain M.A., Bhutto Z.A., Shar A.H., Kakar M.U., Manzoor R., Chao S. (2017). Silymarin: A potent hepatoprotective agent in poultry industry. World’s Poult. Sci. J..

[38] Kumar N., Dr N. (2018). Study on physico-chemical and antioxidant properties of pomegranate peel. J. Pharmacogn. Phytochem..

[39] Jurenka J. (2008). Therapeutic applications of pomegranate (*Punica granatum* L.): A review. Altern. Med. Rev..

[40] Gözlekçi S., Saraçoğlu O., Onursal E., Ozgen M. (2011). Total phenolic distribution of juice, peel, and seed extracts of four pomegranate cultivars. Pharmacogn. Mag..

[41] Guo C., Yang J., Wei J., Li Y., Xu J., Jiang Y. (2003). Antioxidant activities of peel, pulp and seed fractions of common fruits as determined by FRAP assay. Nutr. Res..

[42] Banerjee N., Kim H., Talcott S., Mertens-Talcott S. (2013). Pomegranate polyphenolics suppressed azoxymethane-induced colorectal aberrant crypt foci and inflammation: Possible role of miR-126/VCAM-1 and miR-126/PI3K/AKT/mTOR. Carcinogenesis.

[43] Prakash C.V.S., Prakash I. (2011). Bioactive chemical constituents from pomegranate (*Punica granatum*) juice, seed and peel-a review. Int. J. Res. Chem. Environ..

[44] Opara L.U., Al-Ani M.R., Al-Shuaibi Y.S. (2009). Physico-chemical properties, vitamin C content, and antimicrobial properties of pomegranate fruit (*Punica granatum* L.). Food Bioprocess Technol..

[45] El-Lethey H., Huber-Eicher B., Jungi T.W. (2003). Exploration of stress-induced immunosuppression in chickens reveals both stress-resistant and stress-susceptible antigen responses. Vet. Immunol. Immunopathol..

[46] Huang T.-J., Shirley Li P. (2001). Dexamethasone inhibits luteinizing hormone-induced synthesis of steroidogenic acute regulatory protein in cultured rat preovulatory follicles. Biol. Reprod..

[47] Burger H.G. (1993). Evidence for a negative feedback role of inhibin in follicle stimulating hormone regulation in women. Hum. Reprod..

[48] Decuypere E., Rombauts L., Vanmontfort D., Verhoeven G., Havey S., Etches R.J. (1997). Inhibin from embryo to adult hen. Perspective in Avian Endocrinology.

[49] Li-Chan E.C., Kim H.-O. (2008). Structure and chemical composition of eggs. Egg Bioscience and Biotechnology.

[50] Saki A., Shamsollah T., Ashoori A. (2019). Egg iron enrichment in response to various levels of pomegranate by-product in laying hen diet. Iran. J. Appl. Anim. Sci..

[51] Mateos G.G., Jiménez-Moreno E., Serrano M.P., Lázaro R.P. (2012). Poultry response to high levels of dietary fiber sources varying in physical and chemical characteristics1 1Papers from the Informal Nutrition Symposium, “Exploring Maximum Animal Responses,” were presented at the Poultry Science Association and American Association of Avian Pathologists 2011 Annual Meeting in St. Louis, Missouri, on July 16, 2011. J. Appl. Poult. Res..

[52] Esmaillzadeh A., Tahbaz F., Gaieni I., Alavi-Majd H., Azadbakht L. (2004). Concentrated pomegranate juice improves lipid profiles in diabetic patients with hyperlipidemia. J. Med. Food.

[53] Aengwanich W., Simaraks S. (2004). Pathology of heart, lung, liver and kidney in broilers under chronic heat stress. Pathology.

[54] Kapakin K.A.T., Gümüş R., Halit İ., Kapakin S., Sağlam Y.S. (2012). Effects of ascorbic and α-lipoic acid on secretion of HSP-70 and apoptosis in liver and kidneys of broilers exposed to heat stress. Ank. Üniv. Vet. Fak. Derg..

[55] Koc M., Imik H., Odabasoglu F. (2008). Gastroprotective and anti-oxidative properties of ascorbic acid on indomethacin-induced gastric injuries in rats. Biol. Trace Elem. Res..

